# Multilevel Intervention to Increase Patient Portal Use in Adults With Type 2 Diabetes Who Access Health Care at Community Health Centers: Single Arm, Pre-Post Pilot Study

**DOI:** 10.2196/67293

**Published:** 2025-03-25

**Authors:** Robin Whittemore, Sangchoon Jeon, Samuel Akyirem, Helen N C Chen, Joanna Lipson, Maritza Minchala, Julie Wagner

**Affiliations:** 1 School of Nursing Yale University Orange, CT United States; 2 Department of Epidemiology Richard M. Fairbanks School of Public Health Indiana University Indianapolis, IN United States; 3 School of Public Health Yale University New Haven, CT United States; 4 Department of Behavioral Sciences and Community Health University of Connecticut School of Dental Medicine Farmington, CT United States

**Keywords:** patient portal, mobile phone, diabetes, community health center, adults, diabetic, DM, diabetes mellitus, Type 2 diabetes, T2D, community health centers, CHCs, pilot study, feasibility, self-management, glycemic control, patient portals, social determinants of health, primary outcome, digital health, digital health literacy, health technology, health technologies, psychosocial, efficacy

## Abstract

**Background:**

Diabetes self-management education and support (DSMS) delivered via patient portals significantly improves glycemic control. Yet, disparities in patient portal use persist. Community health centers (CHCs) deliver care to anyone who needs it, regardless of income or insurance status.

**Objective:**

This study aimed to evaluate the feasibility, acceptability, and preliminary efficacy of a multilevel intervention to increase access and use of portals (MAP) among people with type 2 diabetes (T2D) receiving health care at CHCs.

**Methods:**

A within-subjects, pre-post design was used. Adults with T2D who were portal naive were recruited from 2 CHCs. After informed consent, participants met with a community health worker for referrals for social determinants of health, provision of a tablet with cell service, and individualized training on use of the tablet and portal. Next, a nurse met individually with participants to develop a DSMS plan and then communicated with patients via the portal at least twice weekly during the first 3 months and weekly for the latter 3 months. Data were collected at baseline, 3 months and 6 months. The primary outcome was patient activation and engagement with the portal. Secondary outcomes included technology attitudes, digital health literacy, health-related outcomes and psychosocial function.

**Results:**

In total, 26 patients were eligible, 23 received the intervention, and one was lost to follow up. The sample was predominately Latino or Hispanic (17/22, 77%) and reported low income (19/22, 86%< US $40,000/year), low education (13/22, 59% <high school), and no health insurance (12/22, 55%). All participants had access to a Smartphone, but 91% (20/22) had never accessed a health app. The baseline hemoglobin A_1c_ level was 8.31%. Portal activation was high; 100% (22/22) of participants created a portal account and logged in within the first month. Mean participant logins per week over the first 3 months was 3.16 (SD 1.55) and 1.45 (SD 0.93) over the final 3 months; mean logins per month over the first 3 months was 12.65 (SD 6.21) and 5.79 (SD 3.74) over last 3 months. Engagement was high; 96% (20/21) logged in at least twice per month in the first 3 months and 76% (16/21) between 3 and 6 months. At 6 months, improvements were seen in technology confidence, digital health literacy, diabetes self-efficacy, and diabetes distress. Participant satisfaction with MAP was high as was intention to continue portal use. Barriers to clinical integration and recommendations for portal development were identified.

**Conclusions:**

MAP shows promise for improving health equity in portal use for T2D. Larger, controlled studies are needed to determine how best to implement MAP in complex clinical settings and to evaluate efficacy over time.

**Trial Registration:**

ClinicalTrials.gov NCT05180721; https://clinicaltrials.gov/study/NCT05180721

## Introduction

There are significant racial and ethnic disparities in the prevalence, morbidity, and mortality of type 2 diabetes (T2D). Despite medical advances and increased access to medical care, these disparities persist. Racial or ethnic minorities are more likely to have poor glycemic control [1], develop diabetes-related complications [2], and are 1.5-2.3 times more likely to die from diabetes than White individuals [3]. Further, racial and ethnic minorities were particularly affected by COVID-19, with increased risk for infection, morbidity, hospitalization, and mortality [4]. Preexisting conditions, including and especially diabetes, increase the risk for poor COVID-19 outcomes [5,6]. Thus, innovative approaches are urgently needed to address health inequities in T2D.

Patient portals provide secure web-based access to medical records with the capability of messaging providers, filling prescriptions, viewing educational materials, and accessing clinic services. Increased patient portal use has the potential to increase engagement with care and improve diabetes health outcomes. In the general population, patient portal use has been shown to increase office visits while decreasing emergency room visits and hospitalizations [7]. Patient portal use also increases patient knowledge, self-efficacy, decision-making, medication use, and preventive screening [8]. In adults with diabetes, greater portal use of secure messaging with providers led to improved glycemic control (measured based on hemoglobin A_1c_ [HbA_1c_] levels) compared to nonusers [9-12]. Other trials have demonstrated significant reductions in HbA_1c_ through diabetes self-management education or support (DSMS) via the portal [13-15]. Thus, fostering communication with providers and diabetes self-management support are promising features of portals for adults with diabetes.

There is considerable evidence documenting disparities in patient portal use. Adults who are older, Black, Latinx, and those with low socioeconomic status and low health literacy are less likely to use patient portals as an adjunct to clinical care [16,17]. Despite increased access and popularity of patient portal use in the United States with over 92% of health care organizations offering portals in 2015 [18,19], disparities in portal use continue. More than 100 studies have demonstrated substantial disparities in portal use [17]. Concern has been raised that this well-intentioned solution for patient-centered care may actually worsen health inequities unless portal use among the underserved is increased [17,20,21]. Portal adoption interventions can successfully increase portal use. In a systematic review of interventions to increase portal use in vulnerable populations, 67% (12/18) of studies showed a signiﬁcant increase in portal use, predictors of use, or reduced disparities. Free or low-cost internet access, technical training and assistance, and proactive outreach from the health care team through the portal were reported to have the strongest evidence for improving health equities in portal use and outcomes [21]. Technical training was the most effective strategy in improving patient portal logins, use of features, and secure messaging [17]. To date, no interventions on portal adoption have targeted adults with T2D of diverse races or ethnicities with limited resources, who have unique structural and social barriers to portal access, use, and diabetes self-management.

Community health centers (CHCs) play a critical role in addressing health inequities in T2D, providing care to over 27 million people in the United States [22]. The aim of CHCs is to provide affordable, high-quality, comprehensive primary care to medically underserved populations, regardless of insurance status or ability to pay for care. Most CHC patients (92%) who live in poverty or near poverty, as defined by the Federal Poverty Level, are disproportionately from racial or ethnic minority groups (total 63%: 32% Hispanic, 22% Black, 9% other minorities), and have high rates of chronic conditions compared to the general population. In 2018, 21% of adults seen at CHCs had T2D compared to 11% in the general population [23].

Social determinants of health (SDoH) are important considerations in developing interventions for adults with T2D who access care at CHCs. The SDoH Equity framework [24], developed by the World Health Organization (WHO), posits that structural determinants of health operate through intermediary and SDoH to shape health outcomes. Intermediary and social determinants include material circumstances (eg, access to tablets or the internet and food security), behavioral, and biological factors (eg, taking medications), psychosocial factors (eg, technology literacy), and the health care system (access to care). To increase patient portal use in adults with T2D accessing care at CHCs, our intervention goals were (1) to address the intermediary and SDoH by providing tablets, home internet, and technology support and (2) to improve engagement with health care by personalizing care and DSMS through known community health workers and nurses employed at the CHC.

The purpose of this study was to pilot-test a multilevel intervention to increase access and use of portals (MAP). We sought to determine MAP’s feasibility, acceptability, and potential to improve outcomes over 6 months among adults with T2D who access health care at 2 CHCs. Outcomes included portal-related outcomes (portal activation [logins during the first month], portal engagement [logins over 6 months], digital health literacy, technology acceptability), health-related outcomes (HbA_1c_, T2D self-management [medication, blood glucose monitoring, healthy eating, and physical activity], and psychosocial function [diabetes self-efficacy, autonomy support, and diabetes distress]).

## Methods

### Overview

A within-subjects, pre-post design was used to pilot MAP in 22 adults with T2D who were portal naïve. We developed the intervention protocol after seeking feedback from stakeholders on barriers and facilitators to patient portal use and logistics to consider optimizing the implementation of MAP in CHCs [25].

### Recruitment

Participants were recruited from 2 CHCs which have been previously described [25]. The clinics are located in Connecticut which is a small and densely populated state with prominent health disparities. In Connecticut, Black residents are nearly 4 times more likely than white residents to have a diabetes-related lower extremity amputation, and among Latino individuals, the rate is nearly 3 times higher than for non-Latino White individuals [26]. Inclusion criteria for this study were as follows: (1) established patient at 1 of the 2 CHCs, (2) age 21-65 years, (3) diagnosed with T2D >6 months, (4) most recent HbA_1c_ measure >7.5%, (5) no use of the patient portal in the past year, (6) no intention of moving or changing clinic within 6 months, and (7) self-reported ability to read in English or Spanish. Exclusion criteria included cognitive impairment (≥3 incorrect answers on the Six Item Screener) [27] or current gestational diabetes.

Participants were recruited from select primary care provider panels at each clinic. A designated clinic staff member reviewed the weekly schedule for potentially eligible recruits and introduced the study to recruits in order to determine preliminary interest. If interested, a trained research assistant explained the study and determined eligibility with a screening questionnaire. If eligible, an appointment was scheduled in person or via Zoom (Zoom Video Communications) for informed consent, baseline data collection, and study enrollment. Written informed consent and data collection were completed in the language preference of the participant (English or Spanish).

Upon completion of baseline data collection, participants were scheduled for the first intervention session. All participants received standard T2D care at the CHC, which followed the guidelines for T2D management as recommended by the American Diabetes Association [28] (eg, quarterly appointments with primary care providers, medical management, and referrals to specialists as indicated). At the study clinics, trained nurses provided diabetes education individually during clinic appointments, as needed. All participants received the MAP intervention from community health workers (CHWs) and nurses employed at the clinics. Clinics were compensated for the salary of the nurses and CHWs for training, delivering the intervention, and completing study tasks.

### Training and Supervising Interventionists

Before delivering MAP to study participants, CHWs and nurses were provided a 1-day, in-person, interactive training on the study protocol. They were also provided supportive supervision throughout the study (weekly to biweekly). Training covered orientation to research and goals of the study; human subjects protection; protocol and documentation; and team roles, responsibilities, and supervision. Training also covered details of the intervention content through a study manual and specific strategies for working with low-literacy or low-numeracy individuals with diabetes [29]. Such strategies include using the teach-back method; asking open-ended questions; avoiding unclear statements (“your test was positive”); keeping sessions brief; presenting small chunks of information; encouraging practice between sessions; using nonnumerical measures (eg, 1 serving butter=size of the tip of the thumb); using pictures when possible; using plain language (no jargon or acronyms); reducing the reading level of written materials; and, using a friendly tone. In addition, they were trained in principles of autonomy support. Autonomy support refers to a patient’s perception that their health care provider recognizes the person’s personal agency, encourages self-efficacy, and supports their self-care choices [30].

### Intervention

The MAP intervention intentionally and directly intervened on the 4 intermediary determinants of disparities as outlined by the WHO Health Equity framework (material circumstances, psychosocial factors, behavioral and biological factors, and the health care system (Multimedia Appendix 1). All study participants received a tablet (which they were allowed to keep at the end of the study) along with internet access for the 6 months of the study.

The intervention was sequenced to first have CHWs assess SDoH needs using a questionnaire specific to each clinic (each clinic had a slightly different form already in use) and connect the participant to relevant community resources (eg, SNAP benefits). CHWs then provided training on how to gain access to the patient portal and on to mastery of the tablet and portal functionality. In addition to lack of training and lack of encouragement to use the portal, other barriers to patient use of portals addressed by CHWs included doubt about portal usefulness, lost passwords, anxiety about viewing medical information, and privacy concerns [31]. Once the technology training and social determinant referrals were completed, participants were referred to the clinic nurse (Multimedia Appendix 2).

Next, the clinic nurse contacted the participant via the portal to provide DSMS. According to the American Diabetes Association, DSMS is “the support that is required for implementing and sustaining coping skills and behaviors needed to self-manage on an ongoing basis” [32,33]. The nurse initially met individually with study participants to establish rapport, assess diabetes self-management (DSM) behaviors, and develop a DSMS plan collaboratively with the participant. Nurses were asked to communicate with patients via the portal at least twice weekly during the first 3 months and weekly for the latter 3 months, individualizing interactions based on participant needs. In these interactions, nurses assessed challenges and successes with the DSM plan of care and provided education, support, and encouragement. Nurses were also provided with a variety of electronic health education resources that could be sent to participants via the portal. Resources were assembled by the researchers from a thorough review of written and video materials available in English and Spanish for adults with low health literacy.

Characteristics of each CHC required that MAP be integrated into clinic operations in a tailored fashion. First, one CHC did not employ CHWs and their care coordinators (who usually addressed patient social needs) were not available to deliver the MAP intervention. Therefore, at that clinic, the study nurse was trained to complete both CHW and nursing components of MAP. Second, the 2 clinics used different portal platforms. Therefore, training procedures and written materials were designed to be equivalent between, but tailored to, each clinic and its platform.

### Data Collection

Data were collected and managed using REDCap (Research Electronic Data Capture; Vanderbilt University) an National Institutes of Health supported, Food and Drug Administration compliant electronic data capture application for data collection and storage. Data were collected from participants at baseline, 3 months, and 6 months including point-of-care HbA_1c_ values. Research assistants entered data at the time of data collection into the database via tablets or a computer. All participants received a gift card after each wave of data collection—US $40 at baseline, US $40 at 3 months, and US $60 at 6 months. Nurses at the clinics extracted clinical data and information technology specialists at the clinics extracted patient portal data from the electronic health record.

### Measures

#### Overview

Demographics were collected at baseline with questions on age, sex, race or ethnicity, marital status, insurance status, and preferred language (English or Spanish). Participants reported educational attainment and indicated whether they require assistance with reading written health information (never or rarely sometimes or often or always). Participants reported annual household income and also rated their financial strain on a 4-point scale from “We have enough and can save” to “We don’t have enough and we have great difficulties” [34].

#### Clinical Characteristics

BMI data were extracted from the electronic health record with the value most proximal to the date of the baseline assessment. Participants self-reported smoking status, and duration of T2D and completed the Charlson comorbidity index [35] which assesses the presence or absence of 20 common comorbidities.

#### Portal-Related Outcomes

Portal activation and engagement were recorded as the number of portal logins per participant per month. Portal activation was defined as the creation of a patient portal account and the use of the portal in the first month. The portal engagement was defined as ongoing use of the portal over the 6-month study duration. We calculated logins per week and per month. Based on previous studies, we defined portal engagement (consistent portal use) as two or more patient logins per month [36] and calculated the percentage of participants meeting this benchmark. Digital health literacy was measured with 4 items (eg, “I can use apps [like Zoom] on my cell phone, tablet or computer on my own without asking for help from someone else”) on a scale from 1=strongly disagree to 5=strongly agree. Technology acceptability was measured via self-report at baseline, 3 months and 6 months with subscales including ease of use (eg, “I think it will be easy [is easy] to send a message to my provider in the patient portal,” perceived usefulness (“I think that using the patient portal will help me [helps me] understand my diabetes care”) and confidence (eg, “I am confident in my ability to review my health records on the patient portal”) on a scale from 1=strongly disagree to 5=strongly agree [37]. Items were tailored to reference each participant’s respective patient portal (MyChart or Healow) [38,39].

#### Health-Related Outcomes

HbA_1c_ was measured using a fingerstick point of care A1CNow+ (PTS Diagnostics). The validity of this point-of-care assessment of HbA_1c_ has been confirmed through comparisons with clinical laboratory measurements of HbA_1c_ [40,41]. Self-report data included self-management, self-efficacy, perceived autonomy support, and diabetes distress. To assess the reliability of self-report scales, α coefficients were calculated. Self-management was measured by the summary of diabetes self-care activities (eg, diet, medication adherence, blood glucose monitoring, and physical activity). Participants are asked in the last 7 days, how many days did you follow your T2D recommendations for each health behavior (from 0 to 7). Reliability and validity of English and Spanish versions have been established [42,43]. Self-efficacy was measured by the Stanford Diabetes Self-Efficacy Scale, an 8-item scale specific to T2D self-management self-efficacy. Items include confidence in exercise, interpreting blood glucose levels, and following dietary recommendations, with response options from 1=not at all confident to 5=totally confident. Reliability and validity have been established in Spanish-speaking adults [44,45], with an α coefficient of 0.92 in our sample. Perceived autonomy support was measured with the 15-item Health-Care Climate Questionnaire [46], which assesses patients’ perceptions of the degree to which they experience the nurse from the CHC to be autonomy supportive versus controlling (eg, “my diabetes provider encourages me to ask questions”). Response options were on a 5-point Likert scale from 1=strongly disagree to 5=strongly agree and the α coefficient was 0.91 in our sample. Diabetes distress was assessed with the 20-item Problem Areas in Diabetes scale (PAID) [47,48] (eg, “feeling overwhelmed by your diabetes”) with response options on a 5-point Likert scale from 0=not a problem to 4=serious problem. This widely used scale has evidence of strong reliability and validity; the α coefficient was 0.96 in our sample.

Feasibility and acceptability of data collection were determined as a priori based on an established framework [49]. Feasibility data included recruitment (recruits invited vs consented), attrition (number of participants who withdrew from the study or were lost to follow-up), and participant technology access and use (smartphone, computer, or tablet). Treatment fidelity was assessed by calculating percent adherence to session checklists created for the CHWs and nurses respectively. Barriers to clinical integration were documented by the researchers during regular supervision with study nurses and research assistants.

Acceptability data included participant surveys at 3 months on satisfaction with the intervention [50] (eg, “Were the topics in the program important to you?”) and participant-reported therapeutic alliance with the nurse (“How much do you like or trust or have confidence in your nurse?”) [51,52] both rated from 1=not at all to 5=extremely. These questionnaires were modified from published versions to be specific to the MAP intervention protocol. At 6 months, we also asked participants about their intention to continue portal use after study completion (yes or no) and whether they would recommend MAP to a friend (yes or no).

### Ethical Considerations

This study (NCT05180721) was approved by the institutional review board at Yale University (IRB# 2000031753; approved on December 21, 2022). All patients provided informed consent before participating in this study.

### Data Analysis

All data were downloaded from REDCap onto a secure server. Descriptive analyses were performed to assess demographic and clinical characteristics of the sample. Distributions of outcome variables were examined for central tendency and dispersion. We estimated the Cohen *d* effect size of MAP on A1C and tested the statistical significance of the change from baseline using longitudinal models, including generalized linear mixed model (GLMM), a logistic model with random intercept (which incorporates the correlation within repeated measures), and a negative binomial model with random intercept. The coefficients of the categorial time variable (ie, baseline, 3 months, and 6 months) represent the average change of HbA_1c_ at 3 and 6 months from baseline. The GLMM included all participants with data at baseline and at least one postintervention value and missing data was handled using the maximum likelihood approach. The repeatedly measured secondary outcomes were analyzed with the same approach using GLMM, with each outcome analyzed individually. We used the GLMM identify link function for continuous variables, the logit link function for binary variables, and the log link function for count variables. Residuals were assessed for normality assumption and variables were transformed with an appropriate form when the normality assumption did not hold. Log-in data from one participant was excluded due to errors encountered in downloading it from the portal that our IT expert could not resolve.

## Results

### Sample

**Figure 1:**
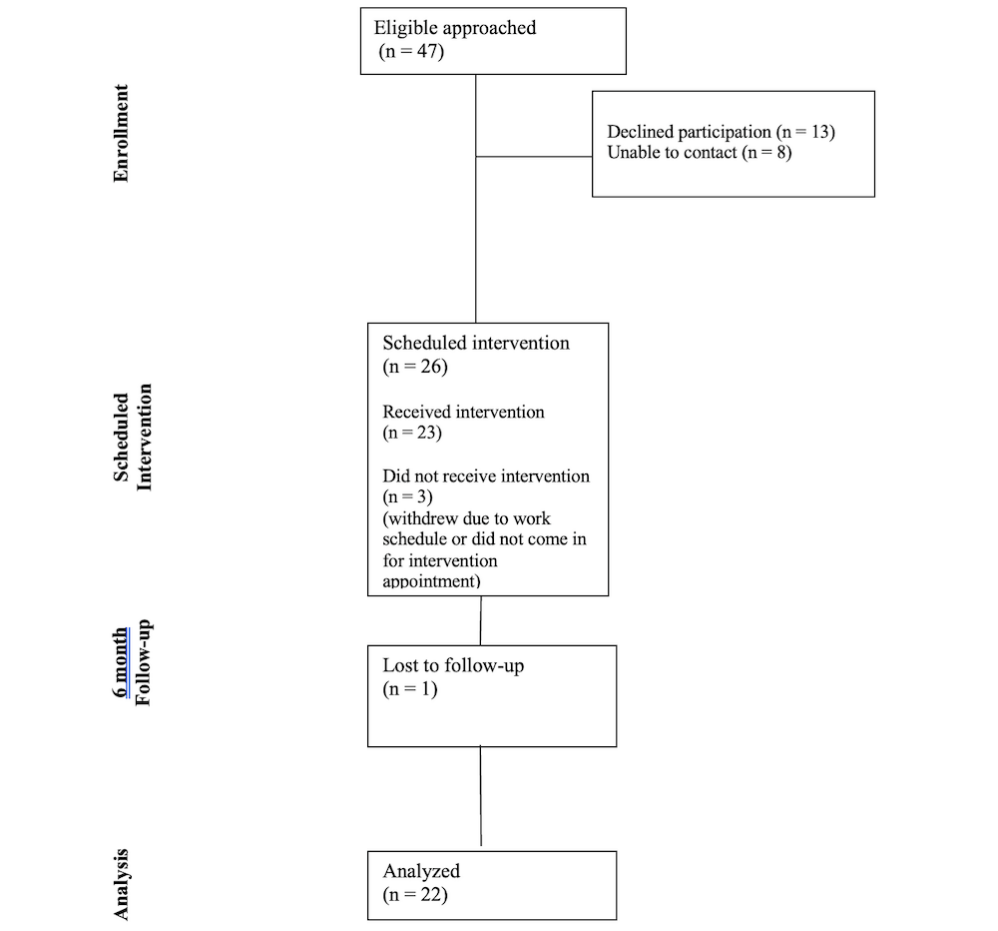
Consolidated Standards of Reporting Trials (CONSORT) diagram.

Recruitment and data collection took place from May 2023 to July 2024. A total of 47 participants were approached about the study, 26 were eligible and provided informed consent, 23 received the intervention and one was lost to follow up (refer to Figure 1 for CONSORT [Consolidated Standards of Reporting Trials] diagram). The sample of completers (n=22) was recruited from 2 clinics, 68% (n=15) from 1 clinic, 32% (n=7) from the other clinic. Refer to Table 1 for demographic characteristics. The sample was predominately Latino or Hispanic (7/22, 77%) had a mean age of 56.32 (SD 10.93) years, 73% (16/22) were female, and 55% (12/22) were married or partnered. The majority reported low-income (86% <US $40,000/year, 19/22), low educational attainment (59%, 13/22 less than high school graduates), and no health insurance (55%, 12/22). All participants had access to a smartphone, but 91% (20/22) had never accessed a health app. Yet, they reported some confidence in using apps, setting up video chats, solving basic technical issues, and using a tablet (mean score 3.1, SD 1.0 with a scale ranging from 1 to 5). They also reported positive perceptions of portal ease of use, usefulness, and confidence in using the portal. The mean duration of diabetes was 11.75 (SD 9.09) years, 36% (8/22) were on insulin at baseline, 14% (3/22) were current smokers, 32% (7/22) reported a history of depression, 59% (13/22) reported severe diabetes distress, 68% (15/22) reported a history of hypertension, and baseline HbA_1c_ was 8.31% (1.05%; measured using the A1CNow system). In total, 73% (16/22) of participants had a baseline HbA_1c_ level greater than the recommended 7.0% for adults with T2D and 73% (16/22) rated their health as fair or poor while 27% (6/22) rated it as good or very good. Mean BMI was 32.21 (SD 5.98), with 23% (5/22) of the sample overweight and 68% (15/22) were categorized as obese. Other baseline clinical data are reported in Table 2 (Data extracted from medical records except for HbA_1c_, which was collected by researchers for study assessment).

**Table 1:** Participant demographics (n=22).

Variables			Values
**Site**			
	Percentage of site 1, n (%)	15 (68)	
	Age in years, mean (SD)	56.32 (10.93)	
**Sex**			
	Percentage of females, n (%)	16 (73)	
	Duration diabetes in years, mean (SD)	11.75 (9.09)	
	Charleston comorbidity index, mean (SD)	3.41 (2.32)	
	Married or partnered (vs not), n (%)	12 (55)	
**Ethnicity**			
	Percentage of Hispanic, n (%)	17 (77)	
**Race, n (%)**			
	Percentage of White	10 (45)	
	Percentage of Black	4 (18)	
	Percentage of other	8; of them, 6 reported Latino/Hispanic (36)	
**Employment, n (%)**			
	Percentage of working part/full time (vs others)	8 (36)	
**Education, n (%)**			
	Percentage of high school graduates or more	9 (39)	
**Annual income,** **n (%)**			
	Less than US $40,000	19 (86)	
**Financial difficulties,** **n (%)**			
	Percentage of difficulties or great difficulties	15 (68)	
**Preferred language, n (%)**			
	Spanish	14 (64)	
	Spanish–not able to converse in English	12 (55)	
**Assistance with reading health information**			
	Sometimes or often or always	10 (46)	
**Health insurance, n (%)**			
	No insurance	12 (55)	
**Self-reported use of community resources, n (%)**			
	Food assistance	7 (32)	
	Medication access	6 (27)	
	Housing	4 (18)	
	Health insurance	4 (18)	
	Transportation	2 (9)	
	Utilities	2 (9)	
	Childcare or employment	0 (0)	
**Home internet stability, n (%)**			
	No home internet or unsure	3 (15)	
	Fair	6 (27)	
	Good	7 (32)	
	Very good	6 (27)	
**Home cell phone data stability, n (%)**			
	Unsure	3 (15)	
	Fair	6 (27)	
	Good	7 (32)	
	Very good	6 (27)	

**Table 2:** Participant clinical characteristics at baseline (n=22).

Variables	Mean (SD)	Recommended values	Meeting recommendations, n (%)
Hemoglobin A_1c_, %	8.31 (1.50)	<7	6 (28)
BMI	32.31 (5.98)	18.5-24.9	2 (9)
Systolic BP^a^, mm Hg	124.57 (14.53)	<130	13 (62)
Diastolic BP, mm Hg	74.48 (7.90)	<80	16 (76)
Cholesterol, mg/dL	161.33 (44.59)	<200	17 (81)
LDL^b^, mg/dL	76.57 (35.73)	<100	16 (78)
HDL^c^, md/dL	53.00 (26.73)	>40	10 (63)
Triglycerides, mg/dL	164.48 (119.24)	<150	15 (71)

^a^BP: blood pressure.

^b^LDL: low-density lipoprotein.

^c^HDL: high-density lipoprotein.

### Protocol Implementation

SDOH needs were assessed before beginning the technology training with 36% (8/22) of participants requiring a referral. Referrals needed were for assistance with utilities (n=4), transportation (n=3), food (n=3), and insurance (n=2). Training on the use of the portal averaged 84.55 (SD 49.32) minutes, with a range between 30-180 minutes. The portal training protocol implementation was high at 85% (524/616 tasks) fidelity across all protocol items and participants. Participants reported moderate confidence in using the portal after the training session with a mean confidence of 2.3 (SD 0.93) on a 3 point-scale (low, moderate, and high confidence). The majority of participants completed the portal training in one session; however, 4 participants (4/22, 18%) required technology support or additional training due to technical challenges using the portal. Nurse protocol implementation was high at 84% (222/264; 12 weeks × 22 participants) at 3 months; but decreased to 52% (137/264) at 6 months with fewer messages sent by nurses over time.

Barriers to integrating MAP into routine clinical care that was noted during regular supervision were varied and included the following: (1) turnover of CHWs, (2) MAP nurses not embedded in the participant’s clinical team, (3) portal or clinic constraints on messaging from patient to PCP, (4) difficulty tracking those portal messages from nurses to patients that were not opened and acknowledged, (5) cumbersome and error-prone portal features for transmitting glucose data from patient to clinician, and (6) few high-quality diabetes education videos suitable for low-literacy, low-numeracy, and Spanish-speaking individuals that could be delivered via the portal.

### Acceptability

Program satisfaction was high at 3 and 6 months (mean 4.03, SD 0.81 and mean 4.14, SD 0.49 on a 5-point scale respectively). All participants indicated that they would recommend the MAP intervention to a friend. Participants also reported high autonomy support from nurses at 3 months which increased at 6 months. At 6 months, 100% (22/22) of participants reported that they would continue to use the portal for diabetes care and 100% (22/22) felt like the portal was helpful for their diabetes care.

### Portal Outcomes

**Figure 2:**
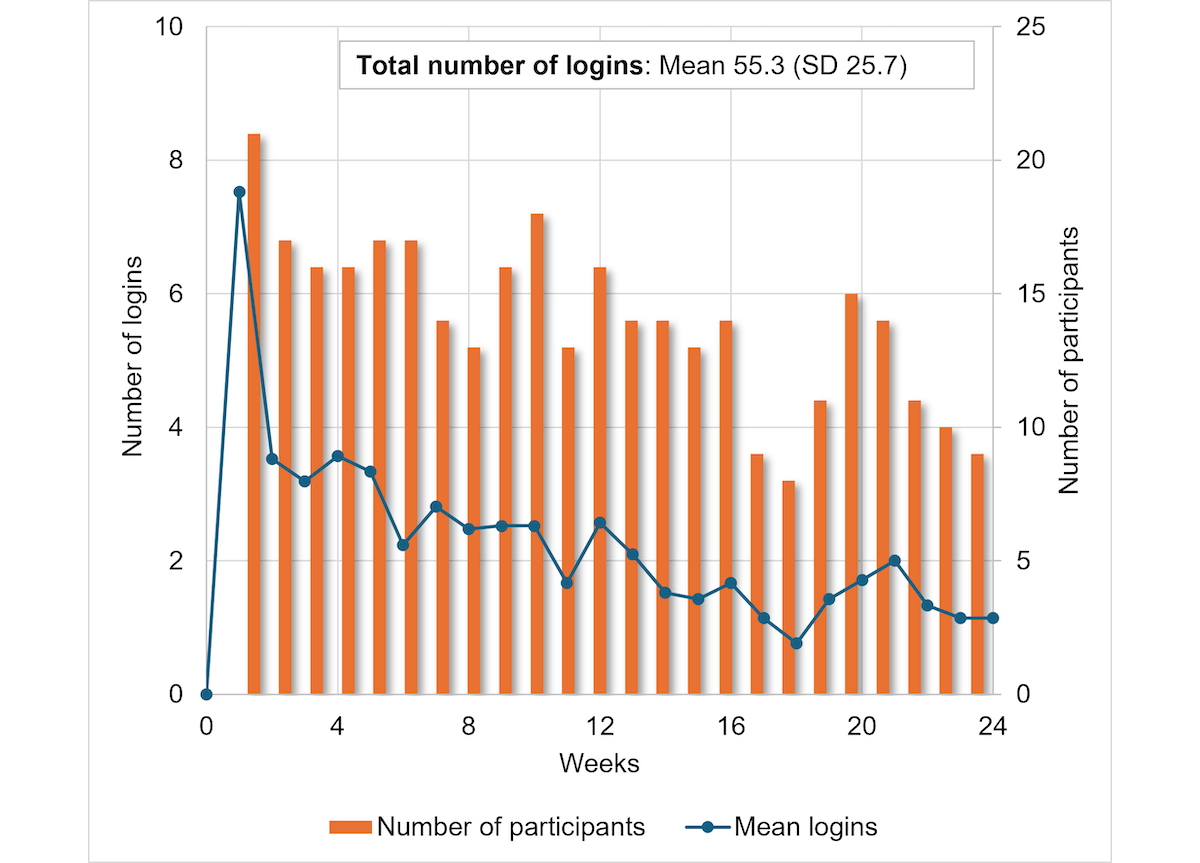
Portal logins over time.

Refer to Table 3 for outcomes over time. Portal activation was high with 100% (22/22) of participants creating a portal account and logging into the portal in the first month (n=21). Mean logins per week was 3.16 (SD 1.55) over the first 3 months and 1.45 (SD 0.93) between 3 and 6 months (Figure 2). Mean portal logins per month were 12.65 (SD 6.21) for the first 3 months and 5.79 (SD 3.74) for the last 3 months. Participant engagement (as defined by the literature) was high, with 96% (20/21) of participants logging into the portal at least twice per month in the first 3 months and 76% (16/21) of participants meeting this benchmark between 3 and 6 months. At baseline, participants perceived that the portal would be easy to use (mean score 4.0, SD 0.55 on a 5-point scale) and useful (mean score 4.25, SD 0.44) with no significant change over time. There was a significant increase in technology confidence over 6 months (*P*<.05), with a trend for increased digital health literacy at 6 months (*P*=.08).

**Table 3:** Change over time for portal and health outcomes (n=22).

			Possible Range		Baseline		3 months		6 months	
**Portal-related outcomes**										
		Portal logins/week^a^	—^b^		—		3.16 (3.18)		1.45 (1.91)	
		Portal logins/month^a^	—		—		12.65 (6.21)		5.79 (3.74)	
		Portal engagement^a^	—		—		96% met benchmark		76% met benchmark	
		Technology confidence	1-5		3.81 (0.86)		4.00 (0.73)		4.38 (0.56)^c^	
		Digital health literacy	1-5		3.15 (1.03)		3.23 (0.93)		3.59 (0.87)^c^	
		Portal perceived ease of use	1-5		4.00 (0.55)		3.89 (0.78)		4.08 (0.67)	
		Portal perceived usefulness	1-5		4.25 (0.44)		4.15 (0.660		4.31 (0.51)	
**Health-related outcomes**										
	Hemoglobin A_1c_, %			—		8.31 (1.65)		8.09 (1.64)		8.23 (1.35)
	PAID^d^			0-100		43.58 (30.51)		29.09 (23.03)^c^		25.45 (24.88)^c^
	DSM^e^ Self-Efficacy			1-5		3.14 (1.01)		3.44 (1.17)		3.68 (1.03)^c^
	Healthcare Climate Questionnaire			1-5		4.15 (0.78)		4.14 (0.83)		3.95 (1.07)
	SDSCA^f^			0-7		—		—		—
	Diet			—		2.82 (2.91)		4.00 (2.54)		3.68 (2.80)
	Exercise			—		2.61 (2.23)		3.23 (2.11)		3.41 (2.29)
	Glucose checking			—		4.66 (2.49)		4.95 (2.44)		5.41 (2.21)
	Footcare			—		5.30 (1.01)		1.06)		1.79 (.94)
	Medication			—		6.65 (1.00)		6.90 (0.31)		6.71 (0.73)
	Use of community resources			0-9		1.32 (1.52)		1.32 (1.55)		1.36 (1.65)

^a^n=21.

^b^Not applicable.

^c^significant change from baseline at a 5% significance level.

^d^PAID: Problem Areas in Diabetes (diabetes distress).

^e^DSM: diabetes self-management.

^f^SDSCA: Summary of Diabetes Self-Care Behaviors; the Healthcare Climate questionnaire measures autonomy support.

### Health Outcomes

In the mixed effect model, diabetes distress (PAID) significantly decreased from baseline to 3 months; it continued to decrease significantly from 3 months to 6 months (*P*<.01; Table 3). The proportion of clinically elevated diabetes distress (ie, PAID>40) was 50% (11/22) at baseline, and it decreased to 36.4% (8/22) at 3 months (*P*=.20) and 22.7% (5/22) at 6 months (*P*=.03). Diabetes self-efficacy increased from baseline to 3 months and continued to increase until 6 months when it became statistically significant. A similar pattern of significant change from baseline to 6 months was found for confidence in using technology and digital health literacy. HbA_1c_ was slightly decreased at 3 months with a small effect size (Cohen *d*=–0.17) but returned to baseline level at 6 months (Cohen *d*=–0.07). There was no significant change in perceived autonomy support or diabetes self-care behaviors.

## Discussion

### Principal Findings

This pilot study evaluated an intervention to increase patient portal use in adults with T2D who access health care at CHCs. We reached a sample of adults of diverse races or ethnicities, low income, and low educational attainment with low use of the patient portal. The MAP intervention was specifically designed to address multiple levels of challenges identified by CHC patients and providers to patient portal usage. Thus, to enhance successful implementation, participants received a tablet and data plan, SDOH screening, training in patient portal navigation with ongoing support, and diabetes education and support by nurses. Overall, feasibility, acceptability, and improvement in portal and health outcomes of clinical significance were demonstrated yet integration into clinical care was challenging.

First, we demonstrated high feasibility by tailoring implementation strategies to meet the unique needs of each clinic. We also demonstrated low attrition, high patient portal activation (22/22, 100%), and high patient portal engagement over 6 months. High participant activation is not surprising as we had dedicated staff available to train participants in portal use and assist with any challenges. Although the measurement of patient portal engagement is not standardized in the literature, a common metric is the frequency of logins per month. Consistent portal use has been defined as two or more patient logins per month according to a recent systematic review of the measurement of patient portal use [36]. In another study, super-users were defined as those logging in twice or more per month [53]. In our study, consistent portal use was demonstrated in 96% of participants over 3 months, 76% of participants between 3 and 6 months, and 87% over a 6-month period among 21 patients. Thus, the results of our portal activation and engagement are encouraging. Factors that likely contributed to these positive outcomes were technology training or support for the duration of the study, participants’ development of a relationship with a clinic nurse, and consistent outreach by the nurse over 6 months via the portal to provide DSMS. While the provision of a tablet and a data plan may have also contributed to portal engagement over time in this study, we judge that increasing access to smartphones combined with free internet sites in community settings (eg, libraries and coffee shops) may be sufficient for future implementation efforts. Decline over time in consistent use may be partially explained by less consistent protocol implementation by nurses from 3 to 6 months and lack of full integration of MAP into clinic workflow.

Engagement over time was supported by improvements in other portal-related outcomes. Whereas there was no change in how participants viewed the portal per se (perceived usefulness and perceived ease of use), participant perceptions of their own interactions with the technology did improve. Increases were observed in both technology confidence (“I am confident in my ability to use MyChart”) and digital health literacy (“I can solve, or figure out how to solve, basic technical issues on my cell phone, computer, or other device”). Those improvements reached statistical significance at 6 months suggesting that individuals may need more than 3 months of supported use of the tablet and the portal to create change in confidence and digital health literacy. Also encouraging was the high level of acceptability including participant satisfaction with the MAP intervention and high intention to continue use of the portal after the MAP study ended.

Second, some clinically important health-related outcomes significantly improved in our small sample. The decrease in diabetes distress at 3 months, with a further decrease at 6 months, is important, given the body of research showing that diabetes distress is robustly associated with lower self-management and may be associated with higher HbA_1c_ [54,55]. The MAP intervention’s reduction in diabetes distress is consistent with findings from other interventions. A meta-analysis of 41 studies testing eHealth interventions for diabetes self-management found that such interventions are effective in reducing diabetes distress [56]. Our rate of elevated diabetes distress (59%) is higher than a meta-analysis of 55 studies, which showed an overall prevalence of 36% of diabetes distress in people with type 2 diabetes, with higher rates in predominantly female samples and those with more comorbidities [57]. Our small sample size, comprised of participants with elevated HbA_1c_ and who enrolled in a treatment study may account for this high rate of distress. Alternatively, diabetes self-management may be exceptionally distressing in the context of high SDoH needs, as is common among patients at CHCs. In this way, MAP may be particularly beneficial for adults with T2D who access health care at CHCs. One study in T2D found that patients who had high diabetes distress at baseline had a greater increase in self-management and a decrease in HbA_1c_ from mHealth DSMS compared to their low-distress counterparts [58].

Diabetes self-efficacy increased at 3 months but only became statistically significant at 6 months, which may reflect our small sample size since larger studies have shown increases by 3 months [59]. Alternatively, this sample with elevated HbA_1c_, high diabetes distress, and high social needs may require 6 months of intervention to increase self-efficacy. Diabetes self-efficacy has been shown in a systematic review to be an important predictor of self-care behaviors [60] including for example medication adherence [61]. The improvement in diabetes self-efficacy observed in our study may prove important for downstream clinical outcomes since interventions that improve self-efficacy have been shown to directly and indirectly improve glycemic control [62].

We observed that HbA_1c_ and DSM improved at 3 and 6 months relative to baseline values but these improvements were not statistically significant in our small sample. Research on the effectiveness of patient portals on glycemic outcomes has produced inconsistent results [13,14,63-65]; yet, observational studies with adequate sample sizes do in fact provide consistent evidence that patient portal use has a beneficial impact on HbA_1c_ [66-68]. The consistency of portal use is also associated with benefits for HbA_1c_. In a study that examined the consistency of patient portal use among adults with T2D (N=95,043), an increasing number of calendar months of patient portal use was associated with a significant decrease in HbA_1c_ levels [69]. In addition, several studies have highlighted the role of patient portals in improving diabetes self-management behaviors. A retrospective cohort study involving over 100,000 adults with diabetes found that when patients are given access to the patient portal via a mobile device, their adherence to oral antihyperglycemic medications improves significantly [66].

In addition to the small sample size, our study may not have produced significant improvements in HbA_1c_ and DSM because of the low degree of integration of MAP into clinical care. We ensured that MAP was delivered in a clinical setting by embedded clinicians, yet we encountered a number of barriers to a deeper level of clinical integration. For example, in one clinic, participants were not able to send portal messages directly to their PCPs because that portal functionality had not yet been activated clinic-wide. As another example, at the other clinic, the MAP nurse was not part of the patient’s own clinical team. Whereas MAP nurses did send monthly progress notes to the patients’ respective PCPs, it is unknown how the PCPs made use of the reports. In our study, one nurse was bilingual in English or Spanish; however, not all were and one participant had Polish as a first language. In these situations, translation of messages was required. Thus, MAP’s good metrics of implementation were only made possible with creative approaches and “work arounds” to clinic and portal idiosyncrasies. It remains untested how effectively MAP could improve glycemic control and DSM when fully integrated into clinical care.

Health care providers and adults with diabetes in CHCs continue to face barriers that limit their use of portals. Well-documented challenges include low health- and technical literacy, lack of regular access to internet-connected mobile phones, limited language concordance between providers and patients, and other SDoH-related issues [25,70]. CHCs also face the challenge of lacking standardized implementation strategies for rolling out portals and supporting patients to use portals. The findings from the current pilot study provide preliminary evidence of what may work in CHC settings to mitigate these barriers and improve portal use among adults with T2D.

### Clinical Implications

Our findings have several clinical implications. First, having clinicians recommend the use of the patient portal and having dedicated staff to provide training and technical support to patients in CHCs can be useful in improving portal use. Racial and ethnic differences in portal use attenuate when clinicians offer portals to all patients irrespective of their racial and ethnic background [71]. In addition, a recent systematic review to summarize research priorities and best practices in patient portals found that studies that report high rates of portal use often have dedicated staff to enroll and assist patients [72]. Evidence-based techniques to educate socially vulnerable patients, such as screening for health literacy, using the teach-back method, and using bilingual messaging, were used in our intervention and can also be adopted by clinicians in CHCs. Many CHCs incorporate nursing case management for patients with chronic conditions. Nurse case management communication could also be expanded to use the patient portal for ongoing check-ins, support, and patient education, which may enhance portal engagement and health outcomes. In a systematic review of the effectiveness of patient education through patient portals, significant improvements in knowledge, self-management, health behaviors, mental health, and select health outcomes were demonstrated [73].

Lastly, clinic administrators at CHCs could work with their electronic health record support team to eliminate the need for email accounts to activate the patient portal. Patients who access care at CHCs may not have email accounts, which creates an additional barrier to portal use among CHC patients [74]. Alternative approaches such as the use of phone numbers to authenticate users should be encouraged, as the majority of patients in CHCs have access to mobile phones.

### Limitations and Future Research

Findings should be interpreted with caution given study limitations, the primary of which is a design without a comparison group or randomization to treatment. Future research should test MAP using a more rigorous study design. Implementation research should identify strategies to more fully integrate interventions like MAP into complex clinical settings. Common metrics of portal use should be used as outcomes, in addition to study-specific indicators, in order to facilitate comparison across studies. Our sample size at each clinic was insufficient to test for differences between clinics in sample characteristics or differences in outcomes. Study samples should also be large enough to be powered to test for clinically meaningful improvements in HbA_1c_ and to include adequate representation of medically underserved individuals so that intervention effects on disparities in portal use—both ameliorating and exacerbating—can be examined. A longer duration of follow-up would allow for investigation of the durability of any treatment effect.

There is also a need for research to develop portal platforms and functionality that better meet the needs of patients and clinicians. This could include, for example, graphical and pictorial data displays, audiovisual capabilities, and libraries of patient education materials. Beyond the patient portal, our preparation for the intervention implementation revealed a paucity of high-quality, patient-friendly diabetes educational materials (pictorial, video, interactive, and gaming) that are designed for low-literacy, low-numeracy, and Spanish-speaking individuals. Whereas a wide variety of materials exist, few if any meet all the above criteria, and those that try tend to be extremely cursory.

### Conclusions

In this pilot study among people with diabetes receiving care at CHCs, the MAP intervention produced high activation and engagement in portal use as well as meaningful improvement in psychosocial outcomes and promising changes in clinical outcomes. Numerous challenges were also identified that can be addressed in future research. Increasing portal use specifically in health disparities populations may be one key to ameliorating disparities in diabetes outcomes.

## Appendix

Multimedia Appendix 1 Multilevel intervention to increase access and use of portals (MAP) components aligned with the WHO (World Health Organization) Health Equity framework.

Multimedia Appendix 2 Sequence and timing of multilevel intervention to increase access and use of portals (MAP).

## Abbreviations

CHC: community health center
CHW: community health worker
CONSORT: Consolidated Standards of Reporting Trials
DSM: diabetes self-management
DSMS: diabetes self-management education and support
GLMM: generalized linear mixed model
HbA_1c_: hemoglobin A_1c_
MAP: multilevel intervention to increase access and use of portals
PAID: Problem Areas in Diabetes scale
REDCap: Research Electronic Data Capture
SDoH: social determinants of health
T2D: type 2 diabetes
WHO: World Health Organization

## Data Availability

The datasets generated or analyzed during this study are available from the corresponding author on reasonable request.
