# Supplementary material for: Multilevel Intervention to Increase Patient Portal Use in Adults With Type 2 Diabetes Who Access Health Care at Community Health Centers: Single Arm, Pre-Post Pilot Study
Source: JMIR Form Res. 2025 Mar 25;9:e67293. doi: 10.2196/67293 (PMC11979536; doi:10.2196/67293)
Supplement: Multimedia Appendix 1 [file formative_v9i1e67293_app1.docx]

**Supplemental Table 1.** MAP Components Aligned with the WHO Health Equity framework

| Intermediary determinant of disparities | Material Circumstance | Psychosocial Factors | Behavioral & Biological Factors | Healthcare System |
| --- | --- | --- | --- | --- |
| MAP intervention component that addresses each intermediary determinant | Provision of tablet, internet access; referral for community resources (e.g., SNAP benefits) | CHW provides literacy and numeracy-informed in-person training and ongoing support for portal use | Nurse provides diabetes self-management support (clinic engagement, medication taking, glucose monitoring, healthy eating) | MAP delivered through existing portal platforms by staff who are embedded at community health centers |
