# Supplementary material for: Multilevel Intervention to Increase Patient Portal Use in Adults With Type 2 Diabetes Who Access Health Care at Community Health Centers: Single Arm, Pre-Post Pilot Study
Source: JMIR Form Res. 2025 Mar 25;9:e67293. doi: 10.2196/67293 (PMC11979536; doi:10.2196/67293)
Supplement: Multimedia Appendix 2 [file formative_v9i1e67293_app2.docx]

**Supplemental Table 2.** Sequence and Timing of MAP.

| Month 0 | Month 1 | Month 2 | Month 3-6 |
| --- | --- | --- | --- |
| Train CHW and nurse in intervention delivery. Train CHW and nurse how to provide autonomy support. Socialize other clinic providers to the intervention. | CHW meets 1:1 with patient in person on 1) how to use the tablet; 2) how to use the portal; 3) social determinants needs assessment; 4) connection to community resources | Nurse contacts patient via portal. Nurse assesses DSM behaviors, co-creates a DSMS plan, provides diabetes self-management education, and refers to ancillary clinic services | CHW remains available to assist patient with tablet, portal and internet use. Nurse continues DSMS. |
